# *IL21R* hypomethylation as a biomarker for distinguishing benign and malignant breast tumours

**DOI:** 10.1080/15592294.2024.2352683

**Published:** 2024-05-09

**Authors:** Zishan Zang, Yifei Yin, Chunlan Liu, Qiang Zhu, Xuandong Huang, Hong Li, Rongxi Yang

**Affiliations:** a Department of Epidemiology, School of Public Health, Nanjing Medical University, Nanjing, China; b Department of Thyroid and Breast Surgery, The Affiliated Huai’an Hospital of Xuzhou Medical University and The Second People’s Hospital of Huai’an, Huaian, China; c Department of Pathology, The Affiliated Huai’an Hospital of Xuzhou Medical University and The Second People’s Hospital of Huai’an, Huaian, China

**Keywords:** Breast cancer, benign breast tumours, differential diagnosis, DNA methylation, IL21R gene, biomarker

## Abstract

Some benign and malignant breast tumours are similar in pathological morphology, which are difficult to be distinguished in clinical diagnosis. In this study, we intended to explore novel biomarkers for differential diagnosis of benign and malignant breast tumours. Methylation EPIC 850K beadchip and RNA-sequencing were used to analyse 29 tissue samples from patients with early-stage breast cancer (BC) and benign breast tumours for differently methylated and expressed genes. The altered methylation of *IL21R* was semi-quantitatively validated in an independent study with 566 tissue samples (279 BC vs. 287 benign breast tumours) using mass spectrometry. Binary logistic regression analysis was performed to evaluate the association between *IL21R* methylation and BC. BC-associated *IL21R* hypomethylation and overexpression were identified in the discovery round. In the validation round, BC patients presented significant *IL21R* hypomethylation compared to women with benign breast tumours (ORs ≥1.29 per-10% methylation, *p-*values ≤ 5.69E–14), and this hypomethylation was even enhanced in BC patients with ER-negative and PR-negative tumours as well as with triple-negative tumours. The methylation of *IL21R* showed efficient discriminatory power to distinguish benign breast tumours from BC (area under curve (AUC) = 0.88), and especially from ER-negative BC (AUC = 0.95), PR-negative BC (AUC = 0.93) and triple-negative BC (AUC = 0.96). We disclosed significant *IL21R* hypomethylation in patients with BC compared to women with benign breast tumours, and revealed the somatic change of DNA methylation could be a potential biomarker for molecular pathology of BC.

## Introduction

Breast cancer (BC) is the most common malignancy in women and the leading cause of cancer-related mortality worldwide [1,2]. According to Global Cancer Statistics 2020, there were about 2.3 million new cases of BC, accounting for 11.7% of all cancer cases [3].

Currently, pathological examination is the gold standard for clinical diagnosis of BC, and core needle biopsy (CNB) is the most common method to acquire tissue samples for pathological evaluation [4]. Although CNB assessment of histological characteristics has a relatively high sensitivity and specificity, it is still challenging to diagnose some benign and malignant breast tumours with similar pathologic morphology, such as atypical duct hyperplasia and in situ lesions, in situ lesions and microinvasion, which leads to certain proportion of misdiagnosis or missed diagnosis [4,5]. In addition, the accuracy of pathological diagnosis results highly depends on the experiences of the pathologists and the quality of pathological films. The shortage of pathologists is a common phenomenon in clinical practice, especially in some developing countries, and it often takes a long time to obtain pathological results after biopsy [6,7]. Therefore, molecular detection techniques are developed to assist the diagnosis [5,8]. Carcinoembryonic antigen (CEA) and cancer antigen 15–3 (CA15–3) are commonly used biomarkers for BC detection, which are usually elevated in advanced stages and have low sensitivity and specificity in the diagnosis of early-stage BC [9,10]. Finding novel sensitive and efficient biomarkers to distinguish BC from benign breast tumours alone or in combination with pathological examination is of great importance.

Epigenetic modification influence gene expression without altering the gene sequence. Epigenetic alterations are associated with the initiation and progression of cancer and are early events in the development of cancer, which may serve as candidate biomarkers for the early detection, prognosis, and targeted therapy of cancer [11,12]. DNA methylation can regulate gene expression by recruiting proteins involved in gene suppression or inhibiting the binding of transcription factors to DNA [13]. It is highly stable, reproducible, easy to obtain and detect, and has high clinical sensitivity and dynamic range [14–16]. Aberrant DNA methylation patterns can silence tumour suppressor genes and activate oncogenes, leading to uncontrolled cell proliferation and tumour formation [17]. In particular, DNA methylation has been found to play a pivotal role in the occurrence and development of BC, and be an early diagnostic marker for BC. For example, Croes *et al*. found a higher proportion of *DFNA5* promoter methylation in BC tissues compared to normal breast tissues [18]. Naghitorabi *et al*. found that there was a significant correlation between *CDH1* promoter hypermethylation and BC progression [19]. Apolónio et al. pointed out that *TERT* hypermethylated oncological region (THOR) hypermethylation is an important epigenetic marker in breast tumorigenesis, representing a promising biomarker for BC diagnosis [20].

However, there are relatively few studies on DNA methylation differences between benign and malignant breast tumours, and most of them are focused on candidate genes with small sample size [21–23]. In this study, to discover and verify BC-associated methylation changes with potential for identifying malignancy, we performed Methylation EPIC 850K beadchip array and RNA-Sequencing in fresh-frozen samples from 18 benign breast tumours and 11 malignant breast tumours, and performed further independent validation using a large number of patients by mass spectrometry.

## Materials and methods

### Study population

This study was approved by the Ethics Committee of Nanjing Medical University. All the recruited participants provided written informed consent.

All BC and benign breast tumour tissue samples were from the Second People’s Hospital of Huai’an. The inclusion criteria for BC patients were: (1) all were diagnosed by pathology; (2) without other cancer history and metastatic cancer from other organs; (3) all were female new cases that have not received radiotherapy or chemotherapy. Patients with benign breast tumours were also confirmed by pathology and matched to patients with BC by age and the year of diagnosis.

In the discovery study, a total of 29 fresh-frozen tissue samples from 11 patients with early-stage BC (stage 0-IIA) and 18 patients with benign breast tumour in 2019. BC (stage 0-IIA)” comprises 2 cases at stage 0, 6 cases at stage I, and 3 cases at stage IIA, all classified as invasive ductal carcinoma. All 18 patients with benign breast tumour were breast fibroadenoma. In the validation study, 279 BC and 287 age-matched benign breast tumour formalin-fixed and paraffin-embedded (FFPE) tissue samples were collected from 2017 to 2020. The median age of the BC and benign breast tumour group in the validation study was 55.0 years old (IQR: 46.0–60.0 years old) and 46.0 years old (IQR: 42.0–50.0 years old), respectively. The detailed clinical information about the BC patients, including tumour subtype, tumour stage, tumour size, lymph node involvement, oestrogen receptor (ER) status, progesterone receptor (PR) status, human epidermal growth factor receptor 2 (HER2) status were described in Table 1.

**Table 1.** Clinical characteristics of BC patients.

Characteristics	Type	n	%
Tumour subtype	Intraductal carcinoma	17	6.1
	Microinvasive ductal carcinoma	28	10.0
	Invasive ductal carcinoma	216	77.4
	Invasive lobular carcinoma	8	2.9
	Others	10	3.6
Tumour stage	Stage 0	17	6.1
	Stage I	104	37.3
	Stage II	125	44.8
	Stage III	31	11.1
	Unknown	2	0.7
Tumour size	Tis	17	6.1
	T1	142	50.9
	T2	113	40.5
	T3&T4	7	2.5
Lymph node involvement	pN0	187	67.0
	pN1	59	21.2
	pN2	19	6.8
	pN3	10	3.6
	Unknown	4	1.4
ER status	ER-negative	67	24.0
	ER-positive	210	75.3
	Unknown	2	0.7
PR status	PR-negative	111	39.8
	PR-positive	166	59.5
	Unknown	2	0.7
HER2 status	HER2-negative	112	40.1
	HER2-positive	164	59.8
	Unknown	3	0.1
Three receptor status	Triple-negative	22	7.9
	Non-triple-negative	255	91.4
	Unknown	2	0.7

Abbreviation: BC, breast cancer; ER, oestrogen receptor; PR, progesterone receptor; HER2, human epidermal growth factor receptor 2; Triple-negative, ER-, PR- and HER2-negative.

### Infinium 850K methylation assay and RNA-Sequencing

**Figure 1.**
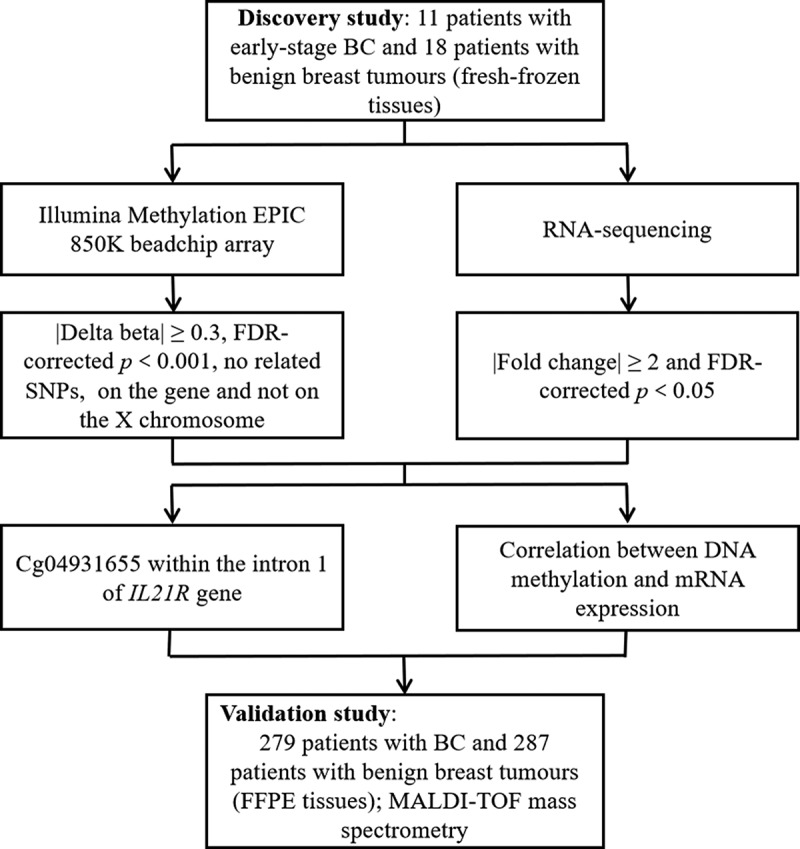
Study design and flow chart. The 29 fresh-frozen tissue samples of the discovery study were subjected to Illumina methylation EPIC 850K beadchip array and RNA-Sequencing. Cg04931655 within intron 1 of *IL21R* gene was selected by comprehensive analysis of binary omics data in a stepwise selection manner. The DNA methylation was well correlated with mRNA expression of *IL21R* gene in fresh-frozen tissue samples during the discovery study. We conducted a further independent validation study with FFPE tissue samples. Binary logistic regression analysis was performed to analyse the correlation between *IL21R* methylation and BC and receiver operating characteristic (ROC) curve analysis was used to verify the ability of *IL21R* methylation level in distinguishing benign and malignant breast tumours.

Abbreviation: BC, breast cancer; FFPE, formalin-fixed and paraffin-embedded; SNP, single nucleotide polymorphism; MALDI-TOF, matrix-assisted laser desorption ionization time-of-flight.

The study design and flow chart were shown in Figure 1. In the discovery study, genomic DNA and total RNA were isolated from 29 fresh-frozen tissue samples by FastPure Blood/Cell/Tissue/Bacteria DNA Isolation Mini Kit (DC112, Vazyme, Nanjing, China) and FastPure Cell/Tissue Total RNA Isolation Kit (RC101, Vazyme, Nanjing, China), respectively, according to the manufacturer’s instructions.

Genome-wide DNA methylation profiles were assayed by Illumina Infinium Human Methylation EPIC 850K beadchip array with single-nucleotide resolution. Probes meeting the following criteria were considered as differentially methylated: (1) methylation difference (|delta beta|) between BC and benign breast tumour groups ≥ 0.3 and FDR-corrected *p*-value < 0.001, (2) CpG site located on gene, (3) no adjacent single nucleotide polymorphisms (SNPs), and (4) CpG site was not located on the X chromosome.

Meanwhile, mRNA expression was measured by next-generation RNA-Sequencing. Initially, RNA quality was evaluated using the Agilent Bioanalyzer 2100 (Agilent Technologies, Santa Clara, CA, USA), and RNA concentrations were measured using the Qubit Fluorometer (Thermo Fisher Scientific, USA). Subsequently, sequencing libraries were prepared as follows: mRNA was purified from total RNA using poly-T oligo-attached magnetic beads, fragmented, and converted into single-strand cDNA, and then synthesized into double-stranded cDNA, which was subjected to end repair, 3’ end A-addition, and ligation of sequencing adapters. The libraries were enriched by PCR amplification of fragments, and sequencing was performed using Illumina NovaSeq 6000 after quality control. Raw reads were trimmed using Fastp to remove adapter sequences, low-quality bases, and reads. Clean reads were aligned to the reference genome (version GRCH38) using HISAT2 with default parameters. Fragments within each gene segment were counted using Stringtie software and then normalized using the trimmed mean of *M* values (TMM) algorithm. The fragments per kilobase of exon model per million mapped (FPKM) value for each gene was used to characterize gene expression. Differences in gene expression levels between benign and malignant breast tissue samples were analysed using the edgeR package. *p*-values were calculated and then corrected using FDR to reduce false positives. Additionally, differential expression fold change (FC) was calculated based on FPKM values. Gene with an absolute expression fold change ≥ 2 and an FDR-corrected *p*-value < 0.05 were considered differentially expressed.

We identified four eligible CpG sites in the 850K methylation beadchip array within the *IL21R* gene by narrowing the position to a range of 1000 bp above and below the transcription start site (Table S1). Among these CpG sites, cg02656594 and cg00050618 were located within the repeat sequence of the gene, rendering them unsuitable for primer design. In addition, the amplicon designed for cg05814654 did not contain the target CpG and was therefore unusable. Thus, we selected CpG site cg04931655, located within intron 1 of the *IL21R* gene, for validation. Specific information regarding these CpG sites has been presented in Table S1.

### MALDI-TOF mass spectrometry

In the validation study, DNA was extracted from the FFPE tissue samples using FastPure FFPE DNA Isolation Kit (DC105, Vazyme, Nanjing, China). Next, the isolated DNA was bisulphite converted by EZ-96 DNA Methylation Gold Kit (D5007, Zymo Research, Orange, USA) according to the manufacturer’s protocol. After bisulphite treatment, all non-methylated cytosine (C) bases in CpG sites were converted to uracil (U), whereas all methylated C bases remained intact. Agena matrix-assisted laser desorption ionization time-of-flight (MALDI-TOF) mass spectrometry (Agena Bioscience, San Diego, California, USA) described by Yang et al. [24] and Yin et al. [25] was then utilized to semi-quantitatively measure DNA methylation levels. In brief, the bisulphite-converted DNA was amplified by bisulphite-specific primers (Table S2). The polymerase chain reaction (PCR) products were incubated with shrimp alkaline phosphatase (SAP) and further treated by T7 transcriptase along with RNase according to the manufacturer’s instructions for Agena EpiTyper Assay. After being cleaned by resin, the final products were distributed into a 384 SpectroCHIP by a Nanodispenser (Agena Bioscience, San Diego, California, USA). The chips were detected by MassARRAY spectrometry, and the quantitative methylation level of each CpG site was collected by SpectroACQUIRE v3.3.1.3 software and visualized by EpiTyper v1.3 software. The EpiTyper v1.3 software automatically calculates methylation levels of each CpG site in the investigated amplicon by comparing the intensities which contained methylated and non-methylated segments. The MassArray generated measurable data for 5 CpG sites in the amplicon of *IL21R* and yielded 5 distinguishable mass peaks. All malignant and benign samples were treated and analysed in parallel throughout the entire processes.

### Statistical analysis

All the statistical analyses were conducted by SPSS Statistical 25.0 and GraphPad Prism (version 8.0). Binary logistic regression analysis was performed to estimate odds ratios (ORs) and their 95% confidence intervals (95% CIs) and establish the predictive model. The covariables were adjusted, including age and different batches for the measurements. Kruskal-Wallis test and Mann-Whitney U test were applied to analyse the significance of the correlation between *IL21R* methylation level and the different clinical characteristics. Comparisons of continuous variables among three or more groups were performed by one-way ANOVA [26–28]. Receiver operating characteristic (ROC) curve analysis was performed to assess the discriminatory power of methylation levels. All statistical tests were two-sided, and *p*-values <0.05 were defined as statistically significant.

## Results

### The discovery of *IL21R* hypomethylation in the tumour tissues of BC

**Figure 2.**
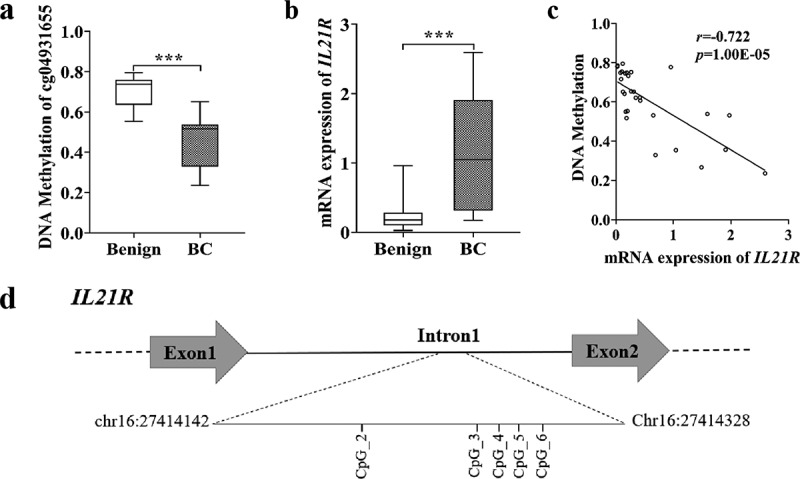
*IL21R* was hypomethylated in BC and negatively correlated with its mRNA expression in BC. (a) Methylation level of *IL21R* (cg04931655) detected by 850K beadchip array were lower in tissue samples from early-stage BC (stage 0-IIA, *n* = 11) than in benign breast tumours (*n* = 18).***p-value < 0.001. (b) mRNA expression levels of the *IL21R* gene measured by RNA-Sequencing were higher in BC than in benign breast tumours. ***p-value < 0.001. (c) mRNA expression levels of *IL21R* were negatively correlated with the methylation levels of cg04931655 evaluated by Spearman’s correlation coefficient. (d) Schematic diagram of *IL21R* amplicon and the locations of the 5 measurable CpG sites (Chr16:27414142 –27,414,328, build GRCh37/hg19, defined by the UCSC Genome Browser). Among them, CpG_2 represented cg04931655.

Based on a comprehensive analysis of differentially methylated and expressed genes in the fresh frozen tissue samples of 18 benign breast tumours and 11 early-stage BC (stage 0-IIA), we identified a CpG site cg04931655 located within the intron 1 of the *IL21R* gene (Figure 1). In 850K methylation beadchip array, cg04931655 showed the most significant methylation difference between benign breast tumour and BC (median methylation value: 0.74 and 0.52 for benign breast tumour and BC, respectively, *p-*value = 2.90E–05; Figure 2a).

Alterations in DNA methylation may affect gene expression. As shown by RNA-sequencing data, the mRNA expression level of *IL21R* gene in BC was significantly higher than that in benign breast tumours (mean FPKM value: 0.23 and 1.15 for benign breast tumour and BC, respectively, *p-*value = 7.49E–04; Figure 2b, Table S3). In addition, the methylation level of cg04931655 was negatively correlated with the expression level of *IL21R* with the Spearman’s correlation coefficient of −0.722 (*p-*value = 1.00E–05; Figure 2c).

### The validation of the association between *IL21R* hypomethylation and BC in FFPE tissues by an independent case-control study

Next, we validated *IL21R* methylation differences between benign and malignant breast tumours in FFPE samples from 287 patients with benign breast tumour and 279 patients with BC. For this, we designed a 187 bp PCR amplicon located on the intron 1 (Figure 2d). The amplicon contained a total of five measurable CpG sites, and CpG_2 represented cg04931655. The methylation levels of all *IL21R* CpG sites were detected successfully by Agena MALDI-TOF mass spectrometry in the FFPE samples.

Binary logistic regression analysis was performed to investigate the association between the methylation of *IL21R* gene and BC. The results showed that hypomethylation of *IL21R* was associated with BC. After adjusted for age and different experimental batches, the ORs per 10% methylation reduction of all CpG sites in the amplicon ranged from 1.29 to 2.03 (all *p-*values ≤ 5.69E–14; Table 2). Among them, the methylation level of CpG_2 showed the most significant difference between benign and malignant breast tumours (OR = 2.03, 95% CI: 1.78–2.32, *p*-value = 1.46E–25; Table 2)

**Table 2.** Methylation difference of *IL21R* between benign breast tumour and BC.

CpG sites	Benign (*n* = 287)	BC (*n* = 279)	OR (95% CI)*	*p-*value*
	Median (IQR)	Median (IQR)	per-10% methylation	
CpG_2	0.67 (0.55–0.72)	0.39 (0.27–0.54)	2.03 (1.78–2.32)	**1.46E–25**
CpG_3	0.84 (0.65–1.00)	0.53 (0.30–0.78)	1.29 (1.21–1.38)	**5.69E–14**
CpG_4	0.71 (0.61–0.77)	0.52 (0.36–0.68)	1.72 (1.52–1.95)	**2.59E–18**
CpG_5	0.80 (0.73–0.86)	0.61 (0.43–0.75)	1.89 (1.65–2.17)	**9.35E–20**
CpG_6	0.79 (0.66–0.86)	0.59 (0.44–0.75)	1.51 (1.36–1.67)	**1.65E–15**

*Logistic regression, adjusted for age and different batches for the measurements. The bold values indicate *p-*value <0.05.

Abbreviation: IQR, interquartile range.

### The correlation between *IL21R* methylation and the clinical characteristics of BC

In order to explore the relationship between *IL21R* methylation levels and the clinical characteristics of BC, we stratified the patients by clinical information and analysed the methylation differences between subgroups by nonparametric Mann-Whitney U test and Kruskal-Wallis test. There was no significant correlation between *IL21R* methylation and tumour subtypes, tumour stage, tumour size or lymph node involvement (all *p*-values > 0.05; Table 3). In spite of that, we observed that the methylation levels of all CpG sites in the *IL21R* amplicon were correlated with ER status, PR status and three receptor status. The methylation levels of each CpG site in the *IL21R* amplicon significantly differed between the ER-positive and ER-negative groups of BC patients (CpG_2: *p*-value = 0.026, CpG_3: *p*-value = 2.42E–04, CpG_4: *p*-value = 2.00E–06, CpG_5: *p*-value = 7.53E–08, CpG_6: *p*-value = 5.30E–05; Table 3). Similarly, differences in methylation levels of each CpG site in the *IL21R* amplicon were also statistically significant in the PR-positive and PR-negative groups (CpG_2: *p*-value = 0.037, CpG_3: *p*-value = 0.001, CpG_4: *p*-value = 4.79E–07, CpG_5: *p*-value = 2.24E–09, CpG_6: *p*-value = 1.00E–06; Table 3). In addition, methylation levels of each CpG site in the *IL21R* amplicon were significantly lower in the triple-negative group compared to the non-triple-negative group of BC patients (CpG_2: *p*-value = 0.014, CpG_3: *p*-value = 0.001, CpG_4: *p*-value = 0.002, CpG_5: *p*-value = 0.001, CpG_6: *p*-value = 0.002; Table 3) Moreover, compared with the HER2-negative group, only CpG_5 showed significantly decreased methylation levels in the HER2-positive group (*p*-value = 0.024; Table 3). In addition, we utilized one-way ANOVA to compare *IL21R* methylation values of different subgroups of tumour subtype, tumour stage, tumour size, or lymph node involvement (Table S4).

**Table 3.** Correlation between *IL21R* methylation and the clinical characteristics of BC.

		Median of methylation levels				
Clinical characteristics	Group (n)	CpG_2	CpG_3	CpG_4	CpG_5	CpG_6
Tumour subtype	Intraductal carcinoma (17)	0.52	0.63	0.57	0.66	0.61
	Microinvasive ductal carcinoma(28)	0.42	0.60	0.52	0.63	0.65
	Invasive ductal carcinoma (216)	0.39	0.51	0.52	0.60	0.58
	*p*-value^a^	0.449	0.216	0.776	0.453	0.102
Tumour stage	Stage 0 (17)	0.52	0.63	0.57	0.66	0.61
	Stage I & II (229)	0.39	0.53	0.52	0.62	0.59
	Stage III (31)	0.44	0.52	0.53	0.59	0.56
	*p*-value^a^	0.251	0.544	0.919	0.627	0.795
Tumour size	Tis (17)	0.52	0.63	0.57	0.66	0.61
	T1&T2 (255)	0.39	0.53	0.52	0.62	0.59
	T3&T4 (7)	0.44	0.40	0.48	0.40	0.62
	*p*-value^a^	0.293	0.533	0.798	0.077	0.772
Lymph node involvement	pN0 (187)	0.38	0.51	0.51	0.59	0.59
	pN1 (59)	0.45	0.64	0.60	0.70	0.61
	pN2&pN3 (29)	0.44	0.54	0.53	0.60	0.56
	*p*-value^a^	0.287	0.256	0.197	0.184	0.900
ER status	ER-negative (67)	0.34	0.40	0.37	0.45	0.47
	ER-positive (210)	0.41	0.59	0.55	0.66	0.63
	*p*-value^b^	**0.026**	**2.42E–04**	**2.00E–06**	**7.53E–08**	**5.30E–05**
PR status	PR-negative (111)	0.37	0.44	0.42	0.50	0.52
	PR-positive (166)	0.42	0.62	0.58	0.67	0.64
	*p*-value^b^	**0.037**	**0.001**	**4.79E–07**	**2.24E–09**	**1.00E–06**
HER2 status	HER2-negative (112)	0.40	0.57	0.53	0.66	0.63
	HER2-positive (164)	0.38	0.52	0.51	0.59	0.57
	*p*-value^b^	0.893	0.119	0.409	**0.024**	0.183
Three receptor status	Triple-negative (22)	0.27	0.35	0.32	0.45	0.41
	Non-triple-negative (255)	0.40	0.57	0.53	0.63	0.60
	*p*-value^b^	**0.014**	**0.001**	**0.002**	**0.001**	**0.002**

^a^Kruskal-Wallis test; ^b^ Mann-Whitney U test. The bold values indicate *p-*value < 0.05.

### Decreased *IL21R* methylation in the BC with ER-negative and PR-negative tumours as well as with triple-negative tumours

**Figure 3.**
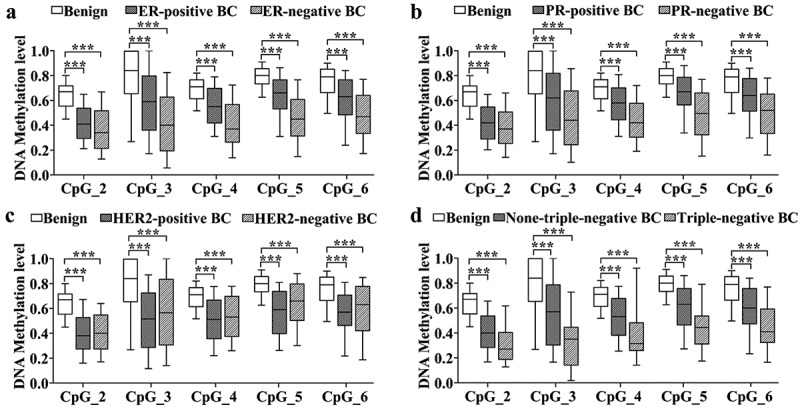
The methylation levels of *IL21R* in BC with different ER, PR, and HER2 status. *IL21R* methylation levels were significantly lower in BC the various ER (a), PR (b) and HER2 (c) statuses compared to the benign breast tumour groups. (d) Both triple-negative (ER-, PR- and HER2-negative) BC and non-triple-negative BC showed hypomethylation of the *IL21R* gene compared to the benign breast tumour groups. The *p*-values were calculated by logistic regression adjusted for age and batches of measurements. ***p-value < 0.001.

We observed the correlation between *IL21R* methylation level and ER, PR, HER2 and three receptor status in BC. Thus, we further analysed the association between *IL21R* methylation and BC stratified by ER, PR, HER2 and three receptor status. *IL21R* hypomethylation was associated with the malignant progression of BC. Compared to benign breast tumours, all five CpG sites in *IL21R* amplicon showed significantly decreased methylation levels in both ER-negative and ER-positive BC patients. Moreover, the methylation level of ER-negative BC patients was lower and the OR value was greater than that of ER-positive BC patients. (all *p-*values ≤ 2.47E–09; Figure 3a, Table S5), Similarly, methylation levels were reduced in both PR-positive and PR-negative BC compared with benign breast tumours, and the reduction was more pronounced in PR-negative BC (all *p-*values ≤ 2.49E–07; Figure 3b, Table S6). Methylation levels of *IL21R* amplicon at all CpG sites were also reduced in both HER2-positive and HER2-negative BC compared with benign breast tumours, but were more significant in HER2-positive BC (all *p-*values ≤7.98E–07; Figure 3c, Table S7). In addition, compared to benign breast tumours, the methylation levels of *IL21R* were significantly lower in BC patients with triple-negative, covariates adjusted ORs were more than 1.54 for per 10% reduction in methylation level of each CpG site (Figure 3d, Table S8).

### *IL21R* methylation as a potential biomarker to distinguish benign and malignant breast tumours

**Figure 4.**
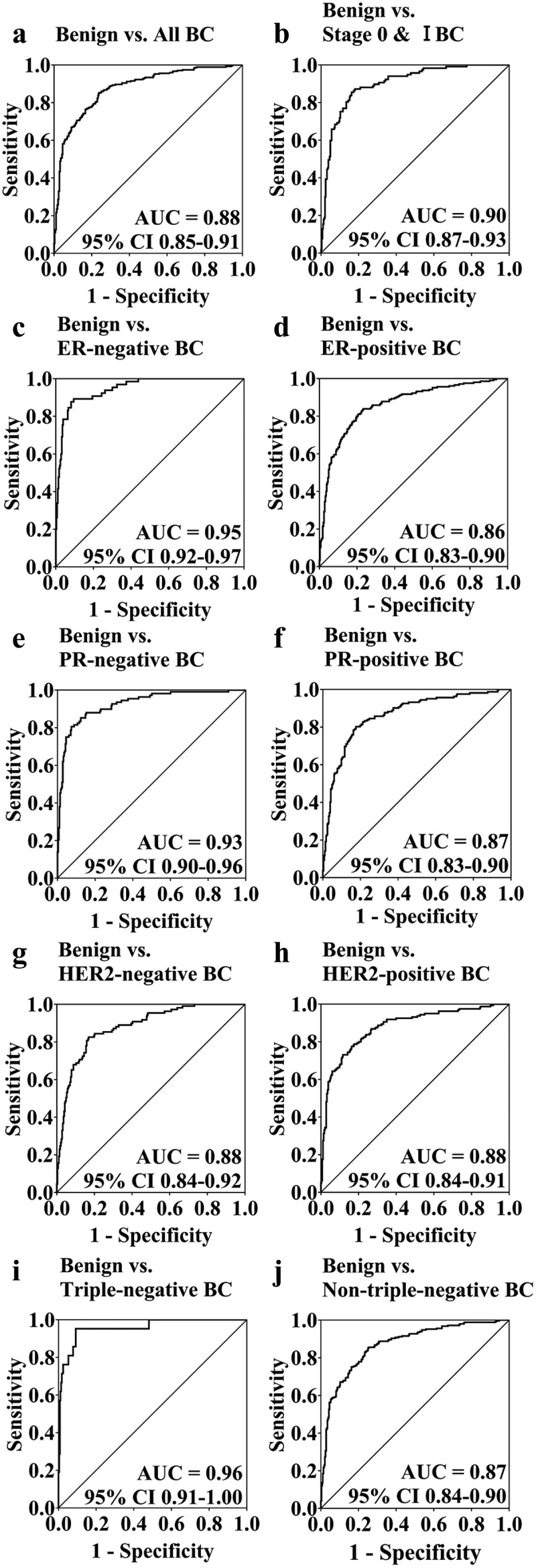
The clinical value of *IL21R* methylation in distinguishing BC cases from patients with benign breast tumour. The methylation levels of five CpG sites within *IL21R* gene were generated a prediction probability. (a) ROC curve analyses for the discriminatory power of *IL21R* methylation to distinguish BC cases from benign breast tumour subjects. (b) ROC curve analyses for the discriminatory power of *IL21R* methylation to distinguish BC cases at very early stage from benign breast tumour subjects. (c-j) ROC curve analyses for the discriminatory power of *IL21R* methylation to distinguish ER-negative (c), ER-positive (d), PR-negative (e), PR-positive (f), HER2-negative (g), HER2-positive (h), triple-negative (i) and non-triple-negative BC cases (j) from benign breast tumour subjects. The ROC analyses were calculated by logistic regression adjusted for age and different batches for the measurements. The grey lines represent the line of no discrimination.

To estimate the potential clinical utility of *IL21R* methylation as a biomarker for the detection of BC, ROC curve analyses were performed adjusted for possible confounding effects by logistic regression. After combining all the five CpG sites in *IL21R* amplicon, we revealed *IL21R* hypomethylation had a good discrimination ability for BC patients [benign vs. BC, area under curve (AUC) = 0.88, 95% CI: 0.85–0.91, Figure 4a]. Further analysis found that the methylation level of *IL21R* showed a more excellent ability to discriminate early-stage BC patients from patients with benign breast tumours (benign vs. stage 0 & I BC, AUC = 0.90, 95% CI: 0.87–0.93, Figure 4b). In addition, we observed that the AUC values of ER-negative, PR-negative, and in particular, triple-negative BC, were significantly increased (benign vs. ER-negative BC: AUC = 0.95, 95% CI: 0.92–0.97; benign vs. PR-negative BC: AUC = 0.93, 95% CI: 0.90–0.96; benign vs. triple-negative BC: AUC = 0.96, 95% CI: 0.91–1.00; Figure 4c, e, i). However, for BC with other receptor status, there was no significant change in the discriminatory power of *IL21R* hypomethylation (AUC values ranged from 0.86 to 0.88; Figure 4d, f, g, h, j).

## Discussion

In recent years, many studies based on peripheral blood from BC patients and normal breast controls have reported DNA methylation alterations as potential biomarkers for BC [29–32]. In the present study, we focus on patients with BC and patients with benign breast tumours, and disclosed altered methylation and expression of *IL21R* gene in the tissue samples by 850K methylation beadchip array and RNA sequencing analysis. Subsequently, *IL21R* hypomethylation in BC was validated in an independent study with FFPE samples of 279 BC and 287 benign breast tumours using mass spectrometry.

*IL21R* is a class I cytokine receptor, mainly expressed in B cells, T cells and NK cells. By binding with IL-21, IL21R activates JAKs-STATs, PI3K-AKT, MAPK, and other signalling pathways [33–35]. *IL21R* plays contrary roles in different cancers. Xue *et al*. observed that compared with healthy controls, the expression levels of *IL21* and *IL21R* were decreased in the serum and lung tissue of lung cancer patients, suggesting *IL21R* as a tumour suppressor in the development of non-small cell lung cancer [36]. Deletion of *IL21R* promoted tumour growth in mouse model of hepatocellular carcinoma (HCC) accompanied by reduced activation and expansion of antitumour lymphocytes, suggesting an anti-tumour role of *IL21R* in HCC [37]. In contrast, *IL21R* was expressed in pancreatic tumour cell lines and in the tissues of pancreatic ductal adenocarcinoma (PDAC), but not in normal pancreatic tissues, indicating that *IL21R* gene might contribute to PDAC progression as an oncogene [38]. *IL21R* may also aggravate colitis and promote colitis-associated colon cancer [39]. Wang *et al*. have reported that *IL21R* is strongly expressed in BC cells MDA-MB-231, and treatment of *IL21* can promote the invasiveness of tumour cells with high expression of *IL21R*, suggesting that *IL21R* may play a carcinogenic role in the occurrence of BC [40]. *IL21R* could mediate the migration and diffusion of BC cells by participating in the MMP signalling pathway [40]. In addition, *IL21R* functioned as an oncogenic factor in gastric cancer, and its high expression was associated with tumour size and lymphatic metastasis, which may represent a potential biomarker for gastric cancer [41]. Here, we revealed the hypomethylation of *IL21R* gene in BC cases, together with a markedly elevated *IL21R* mRNA expression, confirmed the oncogenic role of *IL21R* in BC.

Furthermore, we analysed *IL21R* gene expression in the Tumour Immunity Estimation Resource (TIMER) database (as mentioned by Anuraga et al. [42], https://cistrome.Shinyapps.io/timer/, accessed on 25 January 2024) and The University of Alabama at Birmingham Cancer Data Analysis Portal (UALCAN) database (as mentioned by Anuraga et al. [42], http://ualcan.path.uab.edu, accessed on 25 January 2024). Both databases indicated that *IL21R* expression levels were significantly different between normal and BC tissues, with higher expression levels observed in BC tissues (*p*-value < 0.001) (Figure S1). Our own data from RNA-sequencing further demonstrated that the mRNA expression level of *IL21R* gene in BC was significantly higher than that in benign breast tumours.

Anastasiadi et al. found that methylation within the first intron may regulate gene expression through influencing the interaction between intron enhancers and respective gene promoters [43]. Notably, a majority of the enhancers identified so far are located in intron sequences, and the enhancers can function in either direction. Thus, DNA methylation can impact gene expression by regulating enhancer activity [44,45]. Gene hypomethylation facilitates the activation of new enhancers distributed throughout the gene body, thereby promoting the upregulation of gene expression [46]. Furthermore, it has been proposed that intronic enhancer sequences can bind to transcription factors and that alterations in their methylation status can regulate gene expression by modulating transcription factor binding affinity [47]. Based on the NCBI query results, there are enhancers located near cg04931655 in the *IL21R* gene, which could potentially influence the expression of the *IL21R* gene through intron-mediated enhancement. However, further investigation is required to ascertain the precise mechanism.

BC is a heterogeneous disease. ER status, PR status and HER2 status are all related to the development of BC [48,49]. ER expression is a marker of hormone-dependent breast tumour growth, as well as a predictive and prognostic marker of BC. About 70%-80% of breast tumours are ER-positive. ER-positive BC usually present in the early stages of cancer, with smaller tumours and fewer lymph node metastases, and have a lower risk than ER-negative BC [50]. Several studies have noted that ER-negative breast tumours are associated with a more aggressive phenotype and a poorer prognosis compared to ER-positive breast tumours [49,51]. PR is an oestrogen-regulated gene that is closely related to ER expression, and PR negativity is associated with increased BC growth factor activity and poorer prognosis [52,53]. Overexpression of HER2 can drive tumour growth by activating MAPK and PI3K/AKT signalling pathways, thereby enhancing cell proliferation, invasion, and metastasis [54]. HER2-positive BC tended to exhibit more aggressive behaviour, with increased invasiveness and increased cell motility, leading them to be more likely to recur even after treatment [55]. Previous studies have reported that ER, PR, and HER2 status can affect the DNA methylation status of some genes in BC [56,57]. In our study, the methylation levels of *IL21R* in ER-negative, PR-negative and HER2-positive BC patients were lower than those in ER-positive, PR-positive and HER2-negative BC patients, respectively, and the OR values were higher. Our findings, together with others, suggest the DNA methylation levels of *IL21R* may be related to the malignant progression of BC. Triple-negative BC is a type of BC with negative expression of ER, PR and HER2, accounting for approximately 15%-20% of all BC patients. Compared with other subtypes of BC, patients with triple-negative BC have a higher risk and shorter survival time [58]. We found that *IL21R* methylation was the lowest in triple-negative BC patients, further demonstrating that alterations in DNA methylation were associated with the malignant progression of BC caused by different hormone receptor status.

Currently, databases such as UK Biobank, NCBI GEO, etc. are used for a wide range of cancer-related research [59–61]. Accordingly, we explored the correlation between *IL21R* (cg04931655 refer to CpG_2 in our manuscript) methylation and the clinical characteristics of BC via NCBI GEO database (http://www.ncbi.nlm.nih.gov/geo). The results of GSE148748 (https://www.ncbi.nlm.nih.gov/geo/query/acc.cgi?acc=GSE148748) showed no significant correlation between *IL21R* (cg04931655) methylation and tumour size or lymph node involvement in BC (Table S9), which is in agreement with our results as presented in Table 3 and Table S4.

Paydar *et al*. found that the frequency of *BRCA-1* and *MGMT* promoter methylation in BC patients was significantly higher than that in patients with benign breast tumours. The hypermethylation of *BRCA-1* promoter in patients with benign breast tumours was grouped as high-risk, and the detection of their methylation is helpful for the early diagnosis of BC [21]. In our study, the AUC results showed that the *IL21R* hypomethylation based on FFPE tissue samples has high reliability and accuracy in distinguishing BC patients from subjects with benign breast tumours, especially for BC cases at Stage 0 & I, and BC cases with ER-negative, PR-negative, or triple-negative tumours. These data highlighted the possibility of DNA methylation as a powerful biomarker for the differentiation of benign and malignant breast tumours, as well as for the classification of BC subtypes. The combination of multiple methylation genes as well as different types of biomarkers could even provide sufficient and objective molecular pathological strategies in the future. Nevertheless, validations in multi-centre studies with large samples size are always needed.

Moreover, since the majority of the samples we collected were early-stage BC samples, survival data for Kaplan-Meier analysis were not included. We explored the association between *IL21R* gene methylation and prognosis using the 450K methylation data and survival information from the TCGA database (https://portal.gdc.cancer.gov/, accessed on 7 April 2024). Unfortunately, the 450K methylation data in the TCGA database lacked the CpG site cg04931655. Therefore, we expanded our analysis to include three CpG sites located within 2000 bp above and below cg04931655 for Kaplan-Meier survival analysis. The median methylation value of each CpG site was utilized as a stratification criterion to categorize them into hypomethylated and hypermethylated groups for subsequent survival analyses. However, our analysis revealed that none of the three CpG sites showed statistically significant prognostic differences between the hypermethylation and hypomethylation groups (cg02656594: *p-*value = 0.630, cg05814654: *p-*value = 0.713, cg00050618, *p-*value = 0.083, by Log-rank test; Figure S2). In future studies, we intend to incorporate samples from advanced stages of BC and gather corresponding survival data to conduct a more comprehensive analysis of the relationship between DNA methylation and the prognosis and treatment of BC.

## Conclusion

In summary, our study revealed significantly decreased DNA methylation and elevated mRNA expression of *IL21R* in early-stage BC compared to benign tumours. *IL21R* hypomethylation was strongly correlated with the negative status of ER and PR in BC patients. Taken together, *IL21R* methylation could efficiently distinguish patients with BC, especially triple-negative BC, from women with benign breast tumour, suggesting *IL21R* methylation as a potential biomarker for BC.

## Supplementary Material

-) Clean supplemantary materials.docx

## Data Availability

The datasets generated during and/or analysed during the current study are included in this publication and are available from the corresponding author on reasonable request.
