# Supplementary material for: IL21R hypomethylation as a biomarker for distinguishing benign and malignant breast tumours
Source: Epigenetics. 2024 May 9;19(1):2352683. doi: 10.1080/15592294.2024.2352683 (PMC11086039; doi:10.1080/15592294.2024.2352683)
Supplement: -) Clean supplemantary materials.docx [file KEPI_A_2352683_SM4976.docx]

| Table S1. Information regarding the CpG sites within 1000 bp above and below the *IL21R* transcription start site in the 850K methylation array | | |
| --- | --- | --- |
| probe ID | Delta Beta | *P-*value* |
| cg02656594 | -0.42 | 2.44E-08 |
| cg05814654 | -0.32 | 3.76E-06 |
| cg04931655 | -0.43 | 1.10E-08 |
| cg00050618 | -0.53 | 5.35E-08 |
| * FDR-corrected *P*-value. | | |

| Table S2. The primers and sequence of the *IL21R* amplicon | |
| --- | --- |
| Forward primer | aggaagagagTAGGGGTTTAGTAGTAGGGATGTGG |
| Reverse primer | cagtaatacgactcactatagggagaaggctCACAAAATAAATCCCATCAAACTCT |
| Sequence | TAGGGGCCCAGCAGCAGGGATGTGGCTGAGACGACCCTCTATCTTGAAAACCAGCCTCCAAAATGTAG**CG**GGCTGGGGAAGGAAAGCCAGTTTCAAGCCAAACATAACATGTCAATCACC**CG**ATTCTGGG**CG**GCTGAGT**CG**GGAGGTGA**CG**GGCATGGGTGTAGAGCCTGATGGGACCCATTCTGTG |
| Upper case letters indicated the sequence specific primer regions, and non-specific tags were shown in lower case letters. The EpiTyper assay detected the methylation levels of 5 CpG sites and yielded 5 distinguishable mass peaks. The CpG sites that could be measured are in bold. Cg04931655 is underlined. | |

| Table S3. The mRNA expression of *IL21R* in RNA-Sequencing | |
| --- | --- |
| SampleID | mRNA expression of *IL21R* |
| BC01 | 0.31 |
| BC02 | 1.97 |
| BC03 | 1.59 |
| BC04 | 0.65 |
| BC05 | 0.19 |
| BC06 | 0.69 |
| BC07 | 0.17 |
| BC08 | 2.60 |
| BC09 | 1.91 |
| BC10 | 1.05 |
| BC11 | 1.49 |
| Benign01 | 0.15 |
| Benign02 | 0.09 |
| Benign03 | 0.12 |
| Benign04 | 0.42 |
| Benign05 | 0.08 |
| Benign06 | 0.03 |
| Benign07 | 0.12 |
| Benign08 | 0.10 |
| Benign09 | 0.96 |
| Benign10 | 0.19 |
| Benign11 | 0.27 |
| Benign12 | 0.27 |
| Benign13 | 0.20 |
| Benign14 | 0.42 |
| Benign15 | 0.03 |
| Benign16 | 0.22 |
| Benign17 | 0.16 |
| Benign18 | 0.35 |
| BC-average | 1.15 |
| Benign-average | 0.23 |
| log_2_FC | 2.31 |
| *P*-value* | **7.49E-04** |
| Abbreviation: FC, fold change.  *** FDR-corrected *P*-value. The bold value indicates *P-*value < 0.05. | |

| Table S4. Correlation between *IL21R* methylation and the clinical characteristics of BC | | | | | | |
| --- | --- | --- | --- | --- | --- | --- |
| Clinical characteristics | Group (n) | DNA methylation level（Mean ± SD） | | | | |
|  |  | CpG_2 | CpG_3 | CpG_4 | CpG_5 | CpG_6 |
| Tumor subtype | Intraductal carcinoma (17) | 0.47 ± 0.22 | 0.62 ± 0.31 | 0.54 ± 0.22 | 0.61 ± 0.27 | 0.61 ± 0.22 |
|  | Microinvasive ductal carcinoma (28) | 0.41 ± 0.12 | 0.60 ± 0.33 | 0.54 ± 0.17 | 0.63 ± 0.16 | 0.66 ± 0.17 |
|  | Invasive ductal carcinoma (216) | 0.41 ± 0.21 | 0.52 ± 0.29 | 0.51 ± 0.22 | 0.56 ± 0.24 | 0.55 ± 0.23 |
|  | *P*-value* | 0.460 | 0.212 | 0.659 | 0.335 | **0.045** |
| Tumor stage | Stage 0 (17) | 0.47 ± 0.22 | 0.62 ± 0.31 | 0.54 ± 0.22 | 0.61 ± 0.27 | 0.61 ± 0.22 |
|  | Stage Ⅰ & Ⅱ (229) | 0.40 ± 0.19 | 0.54 ± 0.29 | 0.52 ± 0.21 | 0.59 ± 0.22 | 0.57 ± 0.23 |
|  | Stage Ⅲ (31) | 0.44 ± 0.21 | 0.52 ± 0.31 | 0.51 ± 0.18 | 0.55 ± 0.25 | 0.58 ± 0.22 |
|  | *P*-value* | 0.192 | 0.538 | 0.873 | 0.541 | 0.744 |
| Tumor size | Tis (17) | 0.47 ± 0.22 | 0.62 ± 0.31 | 0.54 ± 0.22 | 0.61 ± 0.27 | 0.61 ± 0.22 |
|  | T1&T2 (255) | 0.40 ± 0.19 | 0.53 ± 0.29 | 0.52 ± 0.21 | 0.58 ± 0.22 | 0.57 ± 0.22 |
|  | T3&T4 (7) | 0.49 ± 0.24 | 0.52 ± 0.36 | 0.49 ± 0.3 | 0.36 ± 0.28 | 0.55 ± 0.35 |
|  | *P*-value* | 0.207 | 0.523 | 0.823 | **0.035** | 0.719 |
| Lymph node  involvement | pN0 (187) | 0.40 ± 0.19 | 0.52 ± 0.30 | 0.51 ± 0.21 | 0.58 ± 0.21 | 0.57 ± 0.23 |
|  | pN1 (59) | 0.43 ± 0.21 | 0.59 ± 0.26 | 0.56 ± 0.20 | 0.61 ± 0.25 | 0.58 ± 0.23 |
|  | pN2&pN3 (29) | 0.45 ± 0.21 | 0.53 ± 0.32 | 0.52 ± 0.18 | 0.56 ± 0.25 | 0.58 ± 0.20 |
|  | *P*-value* | 0.310 | 0.265 | 0.232 | 0.467 | 0.919 |
| *one-way ANOVA analysis. The bold values indicate *P-*value < 0.05. | | | | | | |

| Table S5. Methylation difference of *IL21R* between benign breast tumor and BC stratified by ER status | | | | |
| --- | --- | --- | --- | --- |
| CpG sites | Benign | BC | OR (95% CI)* | *P-*value* |
|  | Median (IQR) | Median (IQR) | per-10% methylation |  |
| 287 benign breast tumors vs. 67 ER-negative BC | | | | |
| CpG_2 | 0.67 (0.55 - 0.72) | 0.34 (0.21 - 0.52) | 2.13 (1.75 - 2.59) | **4.24E-14** |
| CpG_3 | 0.84 (0.65 - 1.00) | 0.40 (0.19 - 0.63) | 1.44 (1.30 - 1.60) | **6.36E-12** |
| CpG_4 | 0.71 (0.61 - 0.77) | 0.37 (0.26 - 0.57) | 2.41 (1.94 - 2.99) | **2.62E-15** |
| CpG_5 | 0.80 (0.73 - 0.86) | 0.45 (0.31 - 0.61) | 2.65 (2.08 - 3.37) | **2.67E-15** |
| CpG_6 | 0.79 (0.66 - 0.86) | 0.47 (0.33 - 0.65) | 1.95 (1.63 - 2.33) | **1.94E-13** |
| 287 benign breast tumors vs. 210 ER-positive BC | | | | |
| CpG_2 | 0.67 (0.55 - 0.72) | 0.41 (0.29 - 0.54) | 2.12 (1.82 - 2.47) | **3.27E-22** |
| CpG_3 | 0.84 (0.65 - 1.00) | 0.59 (0.36 - 0.80) | 1.23 (1.15 - 1.32) | **2.47E-09** |
| CpG_4 | 0.71 (0.61 - 0.77) | 0.55 (0.42 - 0.70) | 1.63 (1.43 - 1.86) | **5.23E-13** |
| CpG_5 | 0.80 (0.73 - 0.86) | 0.66 (0.53 - 0.77) | 1.72 (1.49 - 1.98) | **1.61E-13** |
| CpG_6 | 0.79 (0.66 - 0.86) | 0.63 (0.48 - 0.77) | 1.42 (1.28 - 1.58) | **4.56E-11** |
| * Logistic regression, adjusted for age and different batches for the measurements. The bold values indicate *P-*value < 0.05. | | | | |

| Table S6. Methylation difference of *IL21R* between benign breast tumor and BC stratified by PR status | | | | |
| --- | --- | --- | --- | --- |
| CpG sites | Benign | BC | OR (95% CI)* | *P-*value* |
|  | Median (IQR) | Median (IQR) | per-10% methylation |  |
| 287 benign breast tumors vs. 111 PR-negative BC | | | | |
| CpG_2 | 0.67 (0.55 - 0.72) | 0.37 (0.25 - 0.51) | 2.10 (1.77 - 2.48) | **5.55E-18** |
| CpG_3 | 0.84 (0.65 - 1.00) | 0.44 (0.24 - 0.68) | 1.39 (1.27 - 1.51) | **1.02E-13** |
| CpG_4 | 0.71 (0.61 - 0.77) | 0.42 (0.30 - 0.58) | 2.24 (1.87 - 2.69) | **1.28E-18** |
| CpG_5 | 0.80 (0.73 - 0.86) | 0.50 (0.32 - 0.66) | 2.40 (1.98 - 2.90) | **2.13E-19** |
| CpG_6 | 0.79 (0.66 - 0.86) | 0.52 (0.33 - 0.66) | 1.77 (1.55 - 2.04) | **3.59E-16** |
| 287 benign breast tumors vs. 166 PR-positive BC | | | | |
| CpG_2 | 0.67 (0.55 - 0.72) | 0.42 (0.29 - 0.55) | 2.12 (1.80 - 2.50) | **1.93E-19** |
| CpG_3 | 0.84 (0.65 - 1.00) | 0.62 (0.36 - 0.82) | 1.21 (1.12 - 1.30) | **2.49E-07** |
| CpG_4 | 0.71 (0.61 - 0.77) | 0.58 (0.44 - 0.71) | 1.54 (1.34 - 1.77) | **1.08E-09** |
| CpG_5 | 0.80 (0.73 - 0.86) | 0.67 (0.56 - 0.79) | 1.60 (1.38 - 1.86) | **4.06E-10** |
| CpG_6 | 0.79 (0.66 - 0.86) | 0.64 (0.51 - 0.78) | 1.36 (1.22 - 1.52) | **3.21E-08** |
| * Logistic regression, adjusted for age and different batches for the measurements. The bold values indicate *P-*value < 0.05. | | | | |

| Table S7. Methylation difference of *IL21R* between benign breast tumor and BC stratified by HER2 status | | | | |
| --- | --- | --- | --- | --- |
| CpG sites | Benign | BC | OR (95% CI)* | *P-*value* |
|  | Median (IQR) | Median (IQR) | per-10% methylation |  |
| 287 benign breast tumors vs. 112 HER2-negative BC | | | | |
| CpG_2 | 0.67 (0.55 - 0.72) | 0.40 (0.27 - 0.55) | 2.24 (1.86 - 2.70) | **2.72E-17** |
| CpG_3 | 0.84 (0.65 - 1.00) | 0.57 (0.30 - 0.84) | 1.22 (1.13 - 1.32) | **7.98E-07** |
| CpG_4 | 0.71 (0.61 - 0.77) | 0.53 (0.37 - 0.70) | 1.74 (1.48 - 2.04) | **1.24E-11** |
| CpG_5 | 0.80 (0.73 - 0.86) | 0.66 (0.50 - 0.80) | 1.67 (1.42 - 1.95) | **2.05E-10** |
| CpG_6 | 0.79 (0.66 - 0.86) | 0.63 (0.42 - 0.78) | 1.46 (1.29 - 1.65) | **2.27E-09** |
| 287 benign breast tumors vs. 164 HER2-positive BC | | | | |
| CpG_2 | 0.67 (0.55 - 0.72) | 0.38 (0.27 - 0.53) | 2.00 (1.72 - 2.33) | **9.58E-20** |
| CpG_3 | 0.84 (0.65 - 1.00) | 0.52 (0.28 - 0.73) | 1.32 (1.23 - 1.43) | **4.89E-13** |
| CpG_4 | 0.71 (0.61 - 0.77) | 0.51 (0.35 - 0.67) | 1.81 (1.56 - 2.09) | **1.03E-15** |
| CpG_5 | 0.80 (0.73 - 0.86) | 0.59 (0.40 - 0.74) | 2.02 (1.72 - 2.38) | **9.54E-18** |
| CpG_6 | 0.79 (0.66 - 0.86) | 0.57 (0.46 - 0.71) | 1.56 (1.39 - 1.75) | **8.61E-14** |
| * Logistic regression, adjusted for age and different batches for the measurements. The bold values indicate *P-*value < 0.05. | | | | |

| Table S8. Methylation difference of *IL21R* between benign breast tumor and BC stratified by three receptor (ER, PR, and HER2) status | | | | |
| --- | --- | --- | --- | --- |
| CpG sites | Benign | BC | OR (95% CI)* | *P-*value* |
|  | Median (IQR) | Median (IQR) | per-10% methylation |  |
| 287 benign breast tumors vs. 22 triple-negative BC | | | | |
| CpG_2 | 0.67 (0.55 - 0.72) | 0.27 (0.19 - 0.41) | 2.80 (2.01 - 3.91) | **1.32E-09** |
| CpG_3 | 0.84 (0.65 - 1.00) | 0.35 (0.14 - 0.45) | 1.54 (1.31 - 1.82) | **2.77E-07** |
| CpG_4 | 0.71 (0.61 - 0.77) | 0.32 (0.26 - 0.49) | 2.41 (1.79 - 3.23) | **4.50E-09** |
| CpG_5 | 0.80 (0.73 - 0.86) | 0.45 (0.31 - 0.54) | 2.39 (1.78 - 3.22) | **7.78E-09** |
| CpG_6 | 0.79 (0.66 - 0.86) | 0.41 (0.32 - 0.60) | 1.99 (1.56 - 2.53) | **2.21E-08** |
| 287 benign breast tumors vs. 255 non-triple-negative BC | | | | |
| CpG_2 | 0.67 (0.55 - 0.72) | 0.40 (0.28 - 0.54) | 2.02 (1.76 - 2.31) | **1.26E-23** |
| CpG_3 | 0.84 (0.65 - 1.00) | 0.57 (0.30 - 0.79) | 1.26 (1.18 - 1.35) | **7.16E-12** |
| CpG_4 | 0.71 (0.61 - 0.77) | 0.53 (0.38 - 0.68) | 1.72 (1.52 - 1.96) | **7.85E-17** |
| CpG_5 | 0.80 (0.73 - 0.86) | 0.63 (0.46 - 0.76) | 1.83 (1.59 - 2.10) | **2.39E-17** |
| CpG_6 | 0.79 (0.66 - 0.86) | 0.60 (0.47 - 0.76) | 1.47 (1.33 - 1.63) | **9.63E-14** |
| * Logistic regression, adjusted for age and different batches for the measurements. The bold values indicate *P-*value < 0.05. | | | | |

| Table S9. Correlation between *IL21R* (cg04931655) methylation and the clinical characteristics of BC according to the GEO database | | |
| --- | --- | --- |
| Clinical characteristics | Group (n) | Median/Mean of methylation levels |
|  |  | cg04931655 |
| Tumor size | Tis (3) | 0.14/0.21 |
|  | T1&T2 (73) | 0.26/0.31 |
|  | T3&T4 (6) | 0.33/0.31 |
|  | *P*-value^*^ | 0.268 |
|  | *P*-value^#^ | 0.638 |
| Lymph node  involvement | pN0 (52) | 0.26/0.31 |
|  | pN1 (25) | 0.25/0.27 |
|  | pN2&pN3 (5) | 0.43/0.39 |
|  | *P*-value^*^ | 0.305 |
|  | *P*-value^#^ | 0.280 |
| ^*^ Kruskal-Wallis test.  ^#^ one-way ANOVA analysis. | | |


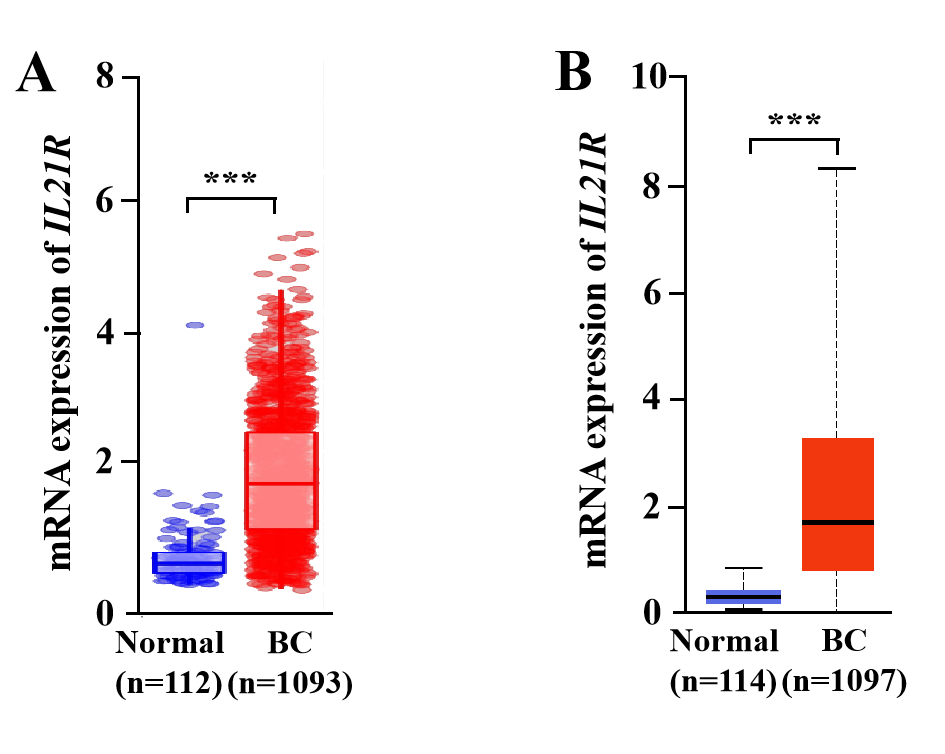


Figure S1. Expression of the *IL21R* gene in breast cancer (BC). (A) Box plot of the *IL21R* gene transcript in normal breast tissues (n = 112) and BC tumors (n = 1093) according to the Tumor Immune Estimation Resource (TIMER) database ([https://cistrome.Shinyapps.io/timer/](https://cistrome.Shinyapps.io/timer/%20) ). Statistical significance was calculated using the Wilcoxon test, ****P-*value < 0.001. (B) Box plot of the *IL21R* gene transcript in normal breast tissues (n = 114) and BC tumors (n = 1097) of The Cancer Genome Atlas (TCGA) data according to the UALCAN database (<http://ualcan.path.uab.edu>). An independent t-test was used to calculate *P*-values, ****P-*value < 0.001.


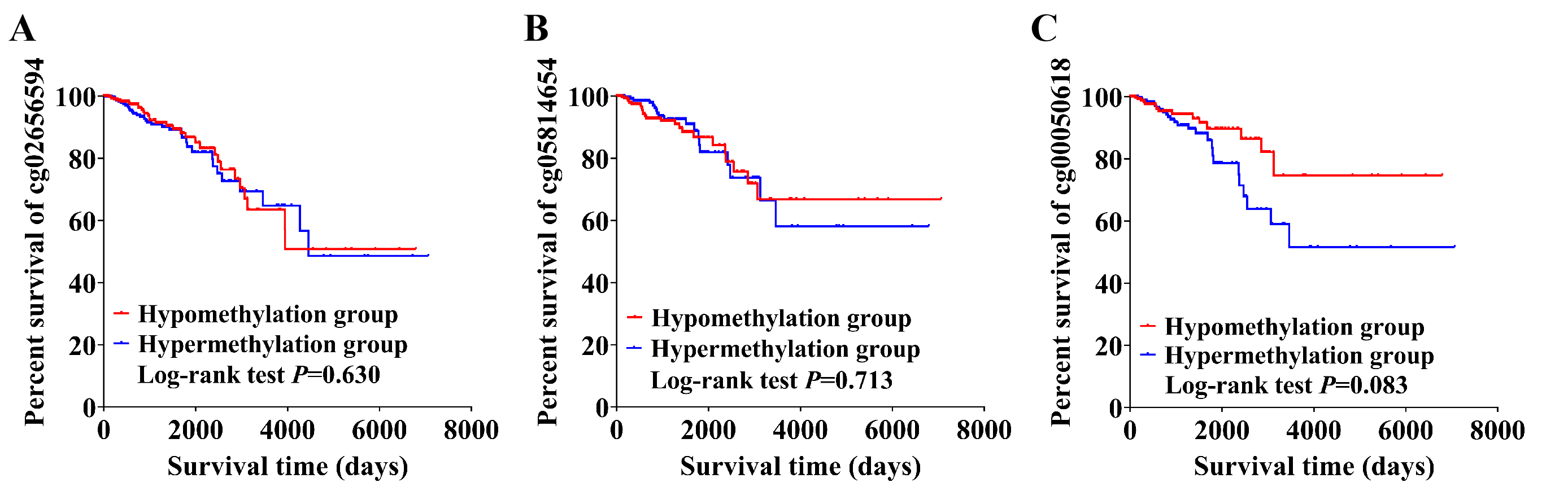


Figure S2. Kaplan-Meier survival curves for hypomethylation and hypermethylation groups by different CpG site of *IL21R*. (A) cg02656594. (B) cg05814654. (C) cg00050618. *P*-values were calculated using the Log-rank test. The association between *IL21R* gene methylation and prognosis was analyzed using the 450K methylation data and survival information from the TCGA database (https://portal.gdc.cancer.gov/, accessed on 7 April, 2024). Due to the lack of CpG site cg04931655 in TCGA database, the analysis was expanded to include three CpG sites located within 2000 bp above and below cg04931655 for Kaplan-Meier survival analysis. The median methylation value of each CpG site was utilized as a stratification criterion to categorize them into hypomethylated and hypermethylated groups for subsequent survival analyses.
